# A novel MIR imaging approach for precise detection of *S. epidermidis* biofilms in seconds

**DOI:** 10.1016/j.bioflm.2025.100270

**Published:** 2025-03-06

**Authors:** Björn van Marwick, Tatyana N. Sevastyanova, Felix Wühler, Barbara Schneider-Wald, Cornelia Loy, Sascha Gravius, Matthias Rädle, Andreas Schilder

**Affiliations:** a Mannheim Technical University, Paul-Wittsack-Straße 10, Mannheim, 68163, Germany; b Department of Orthopaedic and Trauma Surgery, University Medical Centre Mannheim, Medical Faculty Mannheim, University of Heidelberg, Theodor-Kutzer-Ufer 1-3, 68167, Mannheim, Germany

**Keywords:** Staphylococcus epidermidis, (SE), RP62A, (SE+), ATCC 12228, (SE-), Crystal violet, (CV), Extracellular polymeric substances, (EPSs), Mid-infrared, MIR, Fourier-transform infrared (FTIR), Poly-N-Acetylglucosamine, PNAGS

## Abstract

The impact of microbial biofilm growth poses a threat to both human health and the performance of industrial systems, manifesting as a global crisis with noteworthy economic implications for modern society. Exploring new methods and alternative approaches for the detection of biofilm signatures are imperative for developing optimized and cost-effective strategies that can help to identify early-stage biofilm formation. Clinical diagnostic technologies are constantly looking for more affordable, practical and faster methods of prevention and detection of chronic infections in periprosthetic joint infections (PJIs), which are often characterized by biofilm formation on implant surfaces. *Staphylococcus epidermidis (SE)* is especially known for its strong biofilm production and is considered a leading cause of biomaterial-associated infections, including PJIs. Implant-associated infections are severe and difficult to treat, therefore it is crucial to continue identifying bacterial biomarkers that contribute to its structural stability and attachment to implant surfaces. This study presents a pioneering approach for fast spectral detection of biofilm formation with a novel mid-infrared (MIR) scanning system. To highlight the advantages of our MIR system, we performed a comparative analysis with measurements from a commercially available Fourier-transform infrared (FTIR) scanner. We have assessed SE biofilms grown for 3 days comparing the processing times between a commercially available infrared (IR) scanning system (∼8 h/cm^2^), and our innovative scanning approach with rapid self-built MIR detection, achieving a reduction in scanning time to seconds. K-means clustering analysis identified pronounced differences in distribution of clusters, representing a significant variation between biofilm producing (RP62A) and non-biofilm producing (ATCC 12228) bacterial strains. The distribution serves as a critical tool for identifying biofilm phenotypes, particularly where poly-N-acetylglucosamine (PNAG), a key constituent of extracellular polymeric substances (EPS) in *S. epidermidis*, represents the dominant mass fraction in the samples analyzed by our infrared (IR) scanning systems. In addition to faster processing times, our novel MIR system demonstrated significantly higher sensitivity compared to FTIR, enabling clear differentiation between the chemical signatures of biofilm and planktonic strains. The corresponding novel approach integrates advanced data analytics with a newly designed rapid MIR prototype, enabling optimized and swift detection of biofilm signatures. These signatures, now recognized as critical targets in diagnosing complex infections, provide an alternative to traditional microbial detection methods in clinical diagnostics.

## 1 Introduction

Bacterial biofilms exhibit viscoelastic properties and can be viewed as polymer gels, consisting of microbial communities that cluster together and adhere to surfaces [1,2]. Biofilms form a natural barrier and provide chemical and mechanical protection for bacteria from external threats [1]. Its composition can be largely divided into two components: sessile bacterial cells and extracellular polymeric substances (EPS). Both are comprised of proteins, polysaccharides, lipids and DNA materials [3]. However, EPS account for 50–90 % of biofilm biomass and are essential for biofilm functionality [2]. They protect bacteria from the host immune system and external threats, while also serving as a potential source of nutrients [2]. In industrial systems the accumulation of microbial biofilms compromises performance of the technical components and reduces the life span of industrial products by modifying the structure and composition of metal and non-metal surface interfaces [4]. In clinical settings, biofilm-related infections are commonly persistent and challenging to treat, as the biofilm structure offers bacteria significant protection, allowing them to withstand antibiotics [[5], [6], [7]].

Biofilms promote bacterial stability on various surfaces, thereby hindering antibiotic penetration [8,9], and thus the resistance to antimicrobial agents for bacteria within biofilm can be increased up to 1000-fold compared to planktonic bacteria [10], which is explained by limited uptake of antibiotic drugs linked to the strong barrier characteristics of biofilms [9]. *Staphylococcus epidermidis* is particularly recognized for its strong ability to form biofilms and is considered a major contributor to infections associated with implanted medical devices, such as catheters, prosthetic joints, and medical devices [[11], [12], [13], [14]]. The biofilm formation on implant surfaces by *S. epidermidis* typically occurs in three key phases: initial attachment of bacterial cells to the biomaterial, accumulation of bacteria into multiple layers with glycocalyx production, and the development of a mature biofilm [12,15]. Layers of biofilm can attach to the surfaces of orthopedic implants, promoting the survival of pathogens and their aggregation in synovial fluid [13,16]. Furthermore, biofilm-producing bacteria promote more efficient dissemination from implant components to other areas of the body, as they continuously release planktonic cells during the final stage of the biofilm cycle [5,17].

Biomaterial-related infections, including PJI, are some of the most difficult clinical infections to treat [18,19]. Among those infections, *S. epidermidis* have been observed in immunocompromised patients with implants, as well as in cases of prosthetic joint infections (PJIs) and on implant surfaces or bone removed due to aseptic prosthetic loosening [7,[20], [21], [22]]. During revision surgeries, it is common to retrieve implants contaminated with *S. epidermidis*, along with surrounding tissues and bone. The growth of *S. epidermidis* is often seen in low-grade chronic infections, where its strong biofilm formation enhances its pathogenicity [11,23].

Preventing infections from biofilm-forming bacteria requires effective diagnostic tools for early biofilm detection. Modern therapeutic antimicrobial approaches have broadened their focus through targeting EPS and eradication of biofilm [24]. Many current laboratory techniques for detecting biofilm-related infections are indirect and cannot identify the physical presence of biofilm or its structural components, making the diagnosis of chronic infections particularly challenging [21]. For many years, there has been limited progress in enhancing the ability of culture-based and culture-independent methods to distinguish between biofilm-associated and planktonic microorganisms. Sonication offers an innovative approach to PJI diagnostics by disrupting biofilms adhered to prostheses [21,25], potentially allowing the identification of detached EPS through an increase in its mass, making detection more feasible. However, this method has not yet been applied to biofilm detection. As a result, biofilm identification often depends on the microscopic visualization, which is extensively time consuming and not practical in clinical settings [6,25].

Extensive research has been conducted utilizing infrared (IR) technology for the investigation of bacteria and bacterial biofilms [[26], [27], [28], [29]]. However, its application in diagnostics remains complex due to its time-consuming and expensive nature because of measurements at wide spectral ranges. Although IR scanners are typically not applied in diagnostics, they have made a great contribution to scientific research, by revealing the biochemical composition of microorganisms and biofilms and exploring molecular structures of lipids, proteins, nucleic acids, and polysaccharides [[28], [29], [30], [31], [32]].

Despite substantial research being conducted in optimization of infrared (IR) technology for biofilm analysis and characterization, understanding the chemical changes as microbes’ transition from the planktonic state to the biofilm state is crucial. Rapid efficient identification of biofilm markers responsible for its structural stability and its irreversible attachment to implant surfaces could significantly promote innovation in clinical diagnostic technologies and develop cheaper and more practical prevention.

Herein, we employed several valuable methodologies: 1) An optimized method for controlled high-volume biofilm production on silver-coated glass slides, eliminating the need for slide chambers. 2) An innovative imaging approach that combines advanced data analytical tools with a newly designed rapid mid-infrared (MIR) prototype, enabling optimized and swift detection of biofilm signatures—now recognized as a new target in the clinical diagnostics of complex infection cases, offering an alternative to traditional microbial detection. 3) Enhancement of resolution in rapid MIR scanning devices using biofilm-producing and non-biofilm producing *S. epidermidis* as a substrate. 4) The implementation of a novel visualization method for analyzing MIR scanning data.

This study holds significant value for the medical field by demonstrating that targeting specific regions of biofilm spectra allows for faster and more precise detection compared to the broad, time-consuming and expensive spectral analysis traditionally performed by FTIR. However, further work is necessary to implement new data models, which is now feasible with AI learning. Advances in this area of technological innovation, coupled with the evolution of AI in data science, offer promising opportunities for integrating IR spectroscopy into clinical practice, extending its applications beyond research.

## 2 Material methods

### 2.1 Kinetics and viability of bacterial strains

The bacterial strains of *Staphylococcus epidermidis*, RP62A (SE+); ATCC 12228 (SE-); and E*.coli nissle* were stored on beads at −80 °C using a cryopreservation system, Microbank™. Liquid cultures were prepared for each bacterial strain from cryogenically stored bacteria; a single bead was inoculated per 30 ml of Tryptic soy broth (TSB) (Merck Millipore). The kinetics were performed by recording absorbances at optical density of 600 nm (OD_**600**_) using a photometer with a time interval of every 30 min for the first 3 h and every 15 min for the next 9 h. For the quantification of viable cells, the spot plating technique was used to quantify the colony forming units (CFUs) by removing an aliquot from the liquid cultures, in parallel to recordings of OD_**600**_ and performing 7 serial ten-fold dilutions, pipetting 10 μl on divided regions of an agar plate. The number of colonies were counted after 20 h to determine living bacteria. CFU per ml was calculated by (Number of colonies∗dilution factor)/volume of culture plate.

### 2.2 Biofilm cultivation in well plates

Bacterial liquid cultures were prepared from cryogenically stored RP62A and ATCC 12228 in Microbank™; a single bead was inoculated in 30 ml of TSB and incubated for 18 h at 37 °C (150 rpm). The cultures were diluted to an OD_**600**_ between 0.005 and 0.01 to achieve approximately 1.5 million CFU (colony forming units) used for biofilm formation. Biofilms were cultivated in sterile tissue culture-treated polystyrene 24-well plates (ref.: 3526; Costar, USA) at 37 °C (0.1 % CO_2_) for 24 h or 72 h. Growth media was replaced every 24 h in biofilms grown for 72 h.

### 2.3 Quantification of biofilms with crystal violet assay

For verification and comparison purposes, biofilm formation of previously mentioned bacteria strains was quantified by staining with crystal violet (CV) dye (ref.: T123.1; Carl Roth GmbH + Co. KG, Germany). The biofilms were washed with DPBS (ref.: 14040-091; Gibco, Germany) and then fixed with 2 % Formaldehyde (FA) (ref.: P733.1; Carl Roth, Germany). Subsequently, the plates were dried in an oven for 30 min at 50 °C, followed by staining with 0.13 % CV for 10 min. After staining, the plates were washed with DPBS and dried again at 50 °C. The bound CV was solubilized with 30 % acetic acid, and the absorption was measured using 96-well plates (ref.: Z717266; Nunc, Germany) in a microplate reader at OD_**570**_ using TECAN.

### 2.4 Biofilm production on glass slides

Silver-coated glass slides (MirrIR Low-e Corner frosted, Kevley Technologies, USA) were sterilized for 1 h in 100 % ethanol, followed by UV light. The overnight cultures for biofilm formation were diluted to an OD_**600**_ between 0.005 and 0.01. Bacterial biofilms and negative controls were grown for 78 h in TSB on silver-coated glass slides placed inside sterile 100 mm Petri dishes (ref.: 351029; BD Falcon, Germany) in an incubator at 37 °C with 0.1 % CO_2_, with growth media replaced every 24 h. Glass slides were washed twice with PBS and fixed in 2 % FA for 5 min and immediately dried in an oven for 1 h at 50 °C. Dry, labeled samples were sent for infrared spectroscopy and microscopy analysis.

### 2.5 Preparation of samples for IR scanning

Prior to application of IR technology, the biofilm-containing surfaces of FA-fixed silver coated slides were manually marked with a scalpel to generate uniform squares (1 cm^2^) in order to delineate specific areas for analysis. This provided an intrinsic reference for the visible spectra, facilitating straightforward signal classification and precise navigation on the slide surface to have a more accurate idea in the visible range of the measurement locations performed by IR scanning technology. For Fourier-transform infrared (FTIR) imaging examination, the instrument (Spotlight 400, PerkinElmer) required pre-cooling with liquid nitrogen, a process that took approximately 35 min. The sample was manually positioned in the desired measurement area. An overview image, with a measurement time of approximately 20 min per cm^2^, was captured to define the region of interest (ROI) for subsequent spectral measurement. Following subsurface measurement, an area spectral survey was conducted on the sample. Data acquisition was performed with spatial resolution of 6.25 μm, a spectral resolution of 8 cm^−1^ and a travel speed between measurement points of 2.2 m/s. With an accumulation factor of 10, sample acquisition was achieved with a measurement time of approximately 7 h per cm^2^. The spectral measuring range was limited to 750 cm^−1^ to 4000 cm^−1^ per measuring point.

### 2.6 MIR measuring system for rapid scanning of biofilm and bacteria

**Fig. 1:**
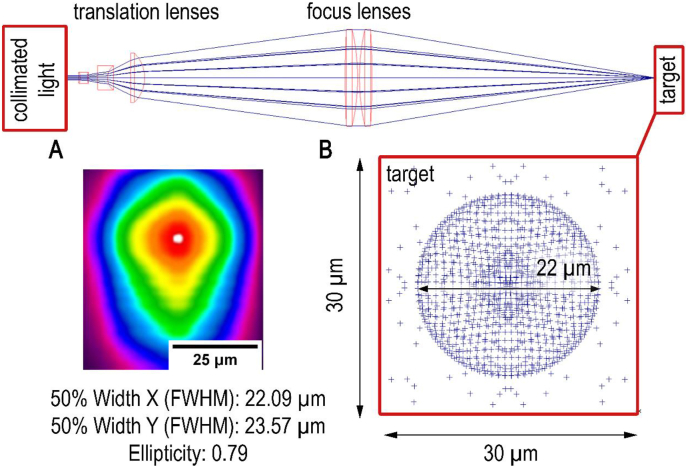
Simulated optical lens structure of the MIR scanner with 50 x 50 beams in a 3 mm radius simulating the input of collimated Laserlight. Optimized for best possible spot size. Comparison of measured (A) and simulated (B) spot size (up to 22 μm) at 3350 cm^−1^.

The newly developed laser-based system (Fig. 1) was tailored for detecting lipid and protein concentrations in substances. Four wavenumbers were employed to assess absorbance and local scattering properties, with scattering used as a reference for absorption measurements [33]. Absorption, directly related to chemical properties, stemmed from the conversion of energy by light beams into molecules through thermal, vibrational, or rotational pathways. Vibrational energy, quantized and molecule-specific, depended on the wavenumber within the range of 12800-200 cm^−1^. When energy was inadequate, rotational conversion occurred, which was also quantized and wavenumber-dependent. Vibrational or rotational movement corresponded to electric charge movement, particularly in groups with dipole moment changes induced by antisymmetric vibration. This movement was targeted by infrared radiation of suitable wavenumbers (***ṽ***) [[34], [35], [36], [37]]. The scanning system's lasers typically targeted the valence vibrations of CH_2_ (2926 cm^−1^), OH stretching vibrations (3200–3600 cm^−1^), and NH (3200–3570 cm^−1^) groups. Lasers (Nanoplus, Germany) were selected at 2790 cm^−1^ (***ṽ***_***1***_) for lipids and 3700 cm^−1^ (***ṽ***_***4***_) for proteins, designated for scattering properties; and at 2926 cm^−1^ (***ṽ***_***2***_) lipids) and 3350 cm^−1^ (***ṽ***_***3***_) proteins) for absorption characteristics. By using one reference laser within the water absorption band and one outside, hydration effects can be assessed, ensuring measurement validity [38]. Confocal detection (VIGO Photonics S.A., Poland) at 2.7 GHz allowed for scanning of 1 cm^2^ at a spatial resolution of 5 μm in less than 12 s for each laser.

Due to optical constraints, the systems resolution has a limitation. The optical structure of the prototype in the focus translator comprises multiple lenses, some adjustable in relation to each other. The translation component compensates for wavenumber-induced diffraction differences and facilitates an easy focus point adjustment by altering lens distance. To computationally determine the potential resolution of the experimental setup, a simulation of the optical system was conducted for Laser 3 (***ṽ***_***3***_) using OptiCAD software. The position of the CAD (Computer Aided Design) models of all lenses was adjusted to achieve a maximally small focal point.

The test setup aimed for a measured spot size of 22 μm, acknowledging the sensitivity of even minor tolerances. A slit-based beam profile measurement using the Ophir NanoScan 2s Pyro/9/5 system was conducted to assess system performance. Fig. 1 depicts a measurement result at ***ṽ***_***3***_, closely approaching the simulated 22 μm under ideal conditions. Variations in spot size were found to be highly dependent on beam profiler settings and positioning accuracy, impacting laser deviations. Laser 3 (***ṽ***_***3***_) achieved the smallest spot size, ranging from 22.09 μm to 23.57 μm. The other lasers were in the range of 22–25 μm with an optical power of 10–12 mW.

To enhance resolution further in mid-infrared scanning, a spatial rastering method was employed, shifting the 20 μm spot by 5 μm steps. This increased the resolution artificially from 20 to 10 μm.

A line grating target (Thorlabs, R1L3S6P) was mounted in the scanning system's object holder to determine achievable resolution. With a 10 μm spatial resolution, structures of up to 50 lines per mm are visualized on the line grating target.

The developed scanning system introduces a novel approach by utilizing narrow spectral bands within a range of strong absorption peaks, an implementation that has not been previously realized in spectral region. For certain sample types, the selection of four specific wavelengths enables chemical clustering results comparable to full-spectrum analyses while significantly reducing acquisition time. By restricting spectral acquisition to a limited number of highly relevant bands, the system achieves measurement speeds up to 225 times faster than conventional FTIR imaging, which requires full-spectrum data collection [39]. Furthermore, the combination of a highly sensitive detector (∼4 × 10^−10^ cm Hz^1/2^ W^−1^) and the application of increased laser power at selected wavelengths enhances spectral contrast for better chemical clustering [38].

### 2.7 FTIR and MIR data (pre-) processing and algorithms

Raw data acquired by Fourier-transform infrared (FTIR) and Mid-Infrared (MIR) imaging techniques yielded initial readouts of light intensities varying between 0 and 65535 counts, which was normalized from 0 to 1, where 1 is the maximal detected intensity (65535 counts), which corresponds to a transmission of 100 %. It is noteworthy that the FTIR system incorporates an automatic background subtraction feature. In the case of our self-built MIR prototype, two references were utilized: a silver-coated slide representing maximum signal and an intrinsic reference within the slide. The scanning system measures both scattering and absorbance at multiple wavelengths, using the local scattering properties of the sample or substrate, with the scattering signals serving as an intrinsic reference. By leveraging scattering as an intrinsic reference, the method compensates for suboptimal background conditions, enabling accurate spectral detection even on rough or uneven surfaces [38]. To address the challenge posed by the occurrence of small pixel offset or image offset in MIR recordings, the set-up was calibrated to the movable microscope stage.

The data acquired with FTIR and MIR were then visualized through a clustering analysis that used the k-means algorithm [40]. The k-means cluster analysis is an unsupervised machine learning approach that is used to make high-dimensional data easier to visualize and interpret by explicitly partitioning each observation [41,42]. The efficacy of this method was demonstrated by its ability to discern distinguishing features associated with biofilm and planktonic phenotypes, which were delineated into five distinct cluster groups (k = 5), each distinguished by a unique color. However, when attempting to differentiate between these various classes, it was observed that two classes alone were insufficient to differentiate between non-biofilm-forming and biofilm-forming bacteria.It is important to note that the measured values were influenced by factors such as the substrate, the cell medium, and manual markings. As a result, the use of multiple classes became necessary to ensure the robustness of the data analysis. As the number of classes increases, assigning chemical interpretations to numerous sub-areas becomes increasingly challenging. Therefore, the study was executed at k = 5, as no informative results could be obtained at k < 5.The study involved 100 iterations of the enhanced center initialization method. For each dataset four wavenumbers (2792 cm^−1^, 2928 cm^−1^, 3352 cm^−1^ and 3704 cm^−1^) were utilized as inputs. The training process was conducted using image patches of 200 × 200 pixels, derived from half of the available dataset. This was performed separately for both datasets (FTIR and MIR). For the mean plots, when a cluster accounted for 10 % of the pixels within a scan, the spectral data from each pixel in that cluster were averaged to generate a representative spectrum. The scatterplots originated from filtered data and cover a subset comprising 10 % of the image data.

### 2.8 Statistics analysis

The statistical analysis of the data generated by biofilm CV assay was performed using GraphPad Prism 10.1.2 for Windows (GraphPad Software Inc., USA). Bar graphs show mean ± SEM. The significance of the data was analyzed using the ratio paired Student's t-test. We considered a two-tailed p-value of less than 0.05 to indicate statistical significance (confidence level 95 %). ns = non-significant and ∗∗∗∗p ≤ 0.001.

For MIR and FTIR data comparisons, Adjusted Rand Index (ARI) and Normalized Mutual Information (NMI) were used to compare the clustering outcomes of data from FTIR with different input wavenumbers. ARI describes the similarity between two clustering results while adjusting for chance. This adjustment allows the Adjusted Rand Index (ARI) to account for the probability of random agreement, with ARI values ranging from −1, indicating no agreement, to 1, indicating perfect agreement [43]. NMI evaluates the similarity between two clustering results by normalizing mutual information. By this NMI provides a value between 0 and 1, indicating no correlation to perfect correlation, respectively [44].

## 3 Results

### 3.1 Standardization of kinetics and growth of biofilm-producing and planktonic bacterial strains

**Fig. 2:**
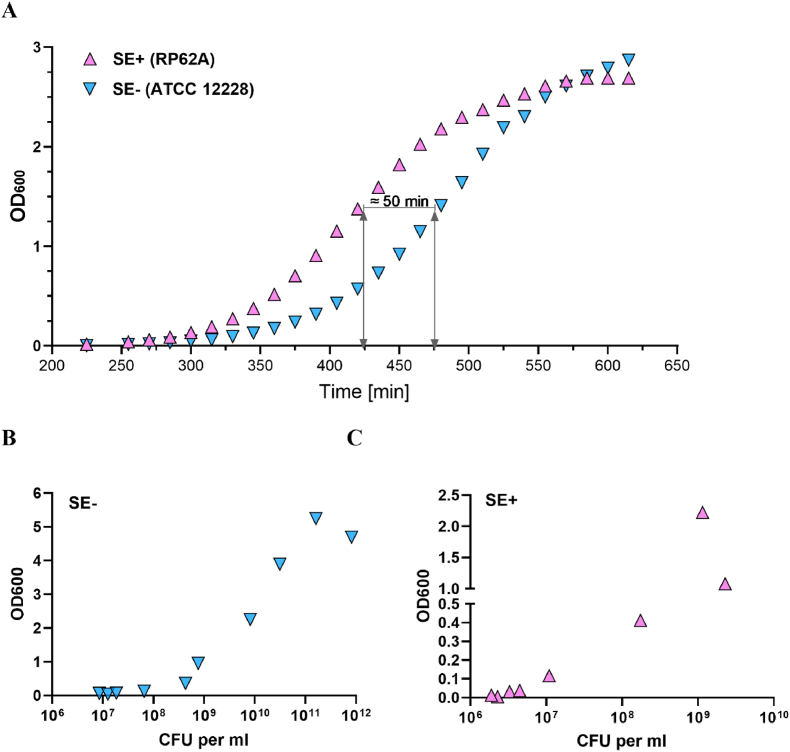
**Kinetics and growth curves of biofilm-producing and planktonic *S. epidermidis.* A.** Kinetics of RP62A (SE+) and ATCC 12228 (SE-) represented by the change in OD_**600**_ absorbances as a function of time. **B.** Growth curves for SE- and C. SE+ were quantified by calculating colony forming unit (CFU)/ml and plotted against OD_**600**_.

For the investigation of biofilms, we used the clinically relevant methicillin-resistant *Staphylococcus epidermidis* strain RP62A (SE+), known for its large biofilm production, high surface adherence, and its frequent association with hospital-acquired infections [11,23]. Firstly, the kinetics and growth dynamics were determined comparing biofilm producing RP62A (SE+) with planktonic strain ATCC 12228 (SE-) (Fig. 2). SE-is considered non-infectious and is not supposed to produce biofilm and can be used as a negative control in biofilm studies [45]. SE + showed higher rate of growth by reaching OD_600_ ∼ 1.5 in ∼430 min, while for SE-it took ∼475min, maximum difference was at OD_600_ ∼ 1.4, which was by 50 min (Fig. 2A).

The stationary phase of SE + growth saturated at OD_600_ ∼ 2.7, however, the stationary phase for SE-was not detected, since the experimental time ran out. SE-required significantly longer time for the stationary phase (Fig. 2A). Growth curves for SE+ and SE-displayed significant differences in the relationship of OD_600_ with CFU/ml. SE-showed maximum OD_600_ ∼ 5, at 8x10^11^ CFU/ml (Fig. 2B), however, SE + displayed maximum OD600–2.2, which was observed at 2x10^9^ CFU/ml (Fig. 2C).

### 3.2 Optimization of biofilm growth

**Fig. 3:**
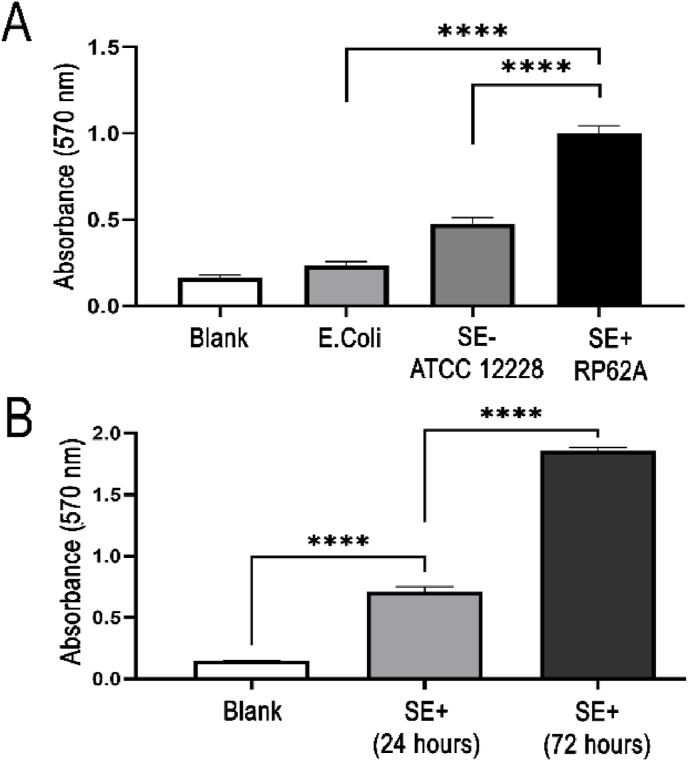
**Quantification of biofilm production via crystal violet staining**. Biofilm growth on polystyrene is depicted by absorbance at 570 nm with TECAN. Blank represents the first negative control prepared from growth media, tryptic soy broth (TSB) in absence of any bacteria. **A.** Quantification of biofilm production by *RP62A* (SE+) after 24 h, compared to negative controls: blank, *E. coli* Nissle, and *ATCC* 12228 (SE-), which are non-biofilm producers. Data are presented as the mean ± SEM of six independent measurements (n = 6) per condition. **B.** Comparison of biofilm growth of *SE +* over 24 and 72 h. Data represent the mean ± SEM from ten independent measurements (n = 10).

Biofilm production was quantified for the biofilm-producing RP62A, *S. epidermidis* (SE+); ATCC 12228, planktonic (SE-); and the low biofilm producer, *Escherichia coli* Nissle 1917 (*E. coli*) as a further control strain. Among these, SE + exhibited the highest absorbance at 570 nm, indicating significantly greater biofilm biomass, with 2.1-fold and 4.2-fold increases compared to SE- and *E. coli*, respectively (P < 0.0001; Fig. 3A). This outcome was expected, as SE+ is well-documented for its robust biofilm formation [11,23]. SE-exhibited a 2.0-fold higher absorbance than *E. coli*, likely due to the retention of adhesion genes in the planktonic SE-strain, despite its lack of biofilm production [45].

In chronic clinical infections, biofilms typically reach full maturity, which is a process that takes longer than 24 h. It has been revealed that after 72 h, *S. epidermidis* fully transitions from the planktonic phase to forming cell clusters, which are characteristic of a mature biofilm [46,47]. To model this more accurately, we extended the incubation period to 72 h for comprehensive biofilm development. Biofilm formation increased by 3.0-fold after 72 h of cultivation compared to 24 h (Fig. 3B).

### 3.3 Characterization of biofilm and planktonic strains with commercial FTIR technology

Rapid detection of biofilm-related infections would be beneficial for clinical applications. It can be achieved by reduction in scanning time, which is one of the major principles in our optimized MIR system that targets reduced number of waves (***ṽ***_***n***_ = 4). To demonstrate that targeting 2790 cm^−1^ (***ṽ***_***1***_), 2926 cm^−1^ (***ṽ***_***2***_), 3350 cm^−1^ (***ṽ***_***3***_) and 3700 cm^−1^ (***ṽ***_***4***_) provides adequate performance in the detection of biofilm, we compared the data acquired using only four wavenumbers; ***ṽ***_***1***_, ***ṽ***_***2***_, ***ṽ***_***3***_ and ***ṽ***_***4***_ with the data of total 10 wavenumbers. This includes these four and additional six: 3450 cm^−1^ (OH), 2878 cm^−1^ (CH), 1655 cm^−1^ (Amide I), 1550 cm^−1^ (Amide II), 1320 cm^−1^ (Amide III), and 1159 cm^−1^ (C–O) [26]. The first measurements were performed with a commercial FTIR scanner, since it has the function of selecting specific wavenumbers in addition to broad range scanning. Adjusted Rand Index (ARI) was employed to assess the agreement between two data partitions represented by cluster analysis comparing ***ṽ***_***n***_ = 4 with ***ṽ***_***n***_ = 10.

**Fig. 4:**
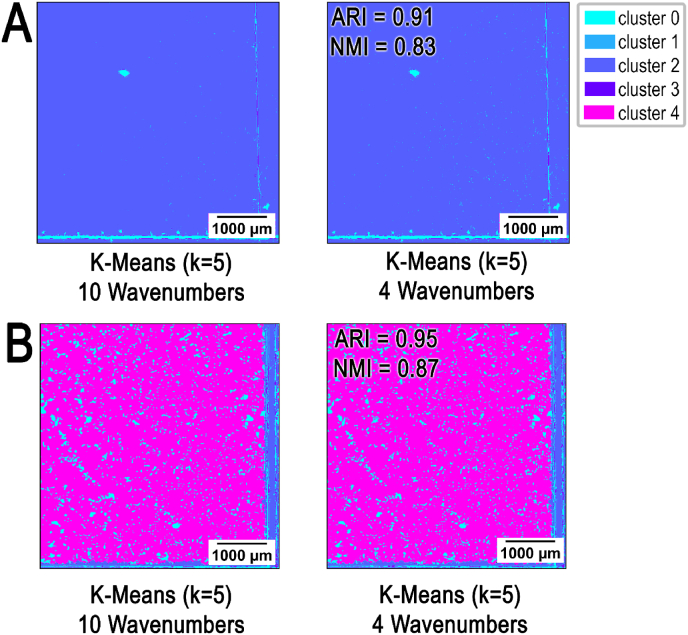
**Evaluation of data concordance in different wavenumber segmentation.***S. epidermidis* (ATCC 12228; SE-; **A**) and *S. epidermidis* (RP62A; SE+; **B**) comparison of analysis of data processed by FTIR of ***ṽ,*** n = 4 versus n = 10 (2790 cm^−1^ (***ṽ***_***1***_), 2926 cm^−1^ (***ṽ***_***2***_), 3350 cm^−1^ (***ṽ***_***3***_), 3700 cm^−1^ (***ṽ***_***4***_), 3450 cm^−1^ (OH), 2878 cm^−1^ (CH), 1655 cm^−1^ (Amide I), 1550 cm^−1^ (Amide II), 1320 cm^−1^ (Amide III), and 1159 cm^−1^) for the segmentation highlighting their accordance showing Normalized Mutual Information (NMI) and Adjusted Rand Index (ARI).

ARI >0.8 indicates high agreement between compared data sets [44,48]. Our data (Fig. 4) passed this threshold value as analysis yielded an ARI of 0.91 for the ATCC 12228 (SE-; Figs. 4A) and 0.95 for the RP62A (SE+; Fig. 4B) datasets**.** These results confirmed that reduced wavenumber set (***ṽ***_***n***_ = 4) meets the required quality standards.

Understanding the chemical composition of bacterial biofilms is crucial. However, spatial resolution of the infrared data must also be addressed, since improved visualization allows precise localization of molecular components within a sample, providing valuable insights into the distribution and organization of biofilm layers.

**Fig. 5:**
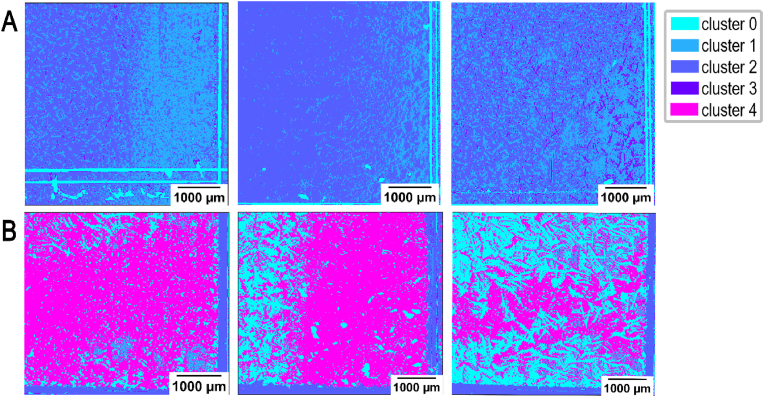
**Visualization of the comparison between biofilm and planktonic states acquired with FTIR scanning.** Four-dimensional (2792 cm^−1^, 2926 cm^−1^, 3352 cm^−1^ and 3704 cm^−1^) input data as 2D arrays acquired with FTIR of SE- (**A**) and SE+ (**B**). A total of three samples (slides) (n = 3) with adhered SE- or SE+ were examined. One clustering image represents one silver coated glass slide processed by FTIR and analyzed by K-means algorithm at convergence.

To visualize the biofilm and planktonic samples processed by FTIR, the four-dimensional dataset was segmented into five clusters (k-value = 5) using the K-Means clustering algorithm [41]. The clustering was influenced by variations in NH and CH concentrations, allowing the scanned data to be categorized into chemically distinct sub-regions. Each of these clusters was represented by a specific color, enabling clear visualization of the distinct chemical domains within the biofilm. This approach effectively highlights the heterogeneity in chemical composition across the biofilm structure (Fig. 5). In the comparison between planktonic (SE-; Fig. 5A) and biofilm (SE+; Fig. 5B) samples there were clear differences detected in their structural architecture. These differences can be easily visualized, which is indicated by variations in color. In biofilm (SE+) samples, the biofilm presence is represented by a magenta color (Fig. 5B).

The data from the FTIR scan was extracted and served as the foundation for the clustering algorithm, which was essential for the future analysis of data obtained from the self-built MIR system. The four wavelengths selected for FTIR scanning differed just by a 2 cm^−1^ shift and were the closest to ***ṽ***_***1***_, ***ṽ***_***2***_, ***ṽ***_***3***_ and ***ṽ***_***4***_ that we could achieve: 2792 cm^−1^ (***ṽ***_***1∗***_), 2928 cm^−1^ (***ṽ***_***2∗***_), 3352 cm^−1^ (***ṽ***_***3∗***_) and 3704 cm^−1^ (***ṽ***_***4∗***_).

**Fig. 6:**
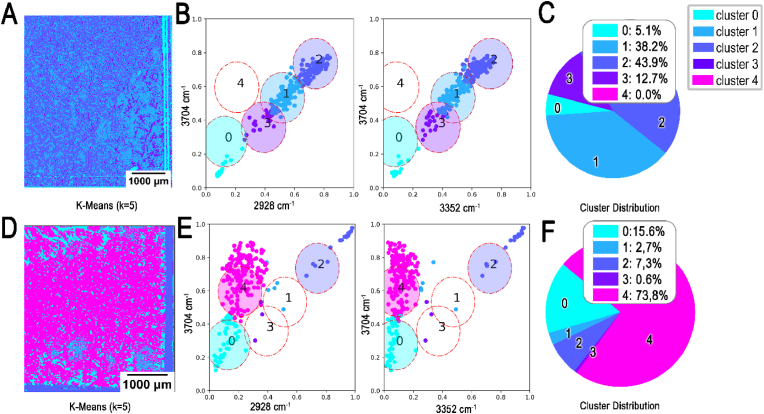
**K-means cluster analysis of biofilm and non-biofilm *S.epidermidis* samples processed by FTIR**. K-means algorithm (k = 5) applied on SE- (**A, B, C**) and SE+ (**D, E, F**). Four-dimensional (2792 cm^−1^, 2928 cm^−1^, 3352 cm^−1^ and 3704 cm^−1^) input data as 2D arrays acquired with FTIR representing one selected clustering image obtained by K-means algorithm at convergence (**A, D**), two corresponding scatterplots for clustering visualization showing 10 % of the data (**B, E**) and a percentage distribution of the data within the cluster analysis (**C, F**).

To quantify the differences between biofilm and planktonic samples scanned by FTIR, the data was processed using cluster analysis, which is depicted by scattering plots (Fig. 6). Scatter plots were generated based on the results of cluster analysis performed on two selected images: one representing a planktonic sample (Fig. 6A) and the other representing a biofilm sample (Fig. 6B). Notably, cluster 4 (Fig. 6B–C and E-F), represented by the magenta color, is found exclusively in biofilm (SE+) samples, with a distribution of 73.3 % (Fig. 6E and F). This cluster is absent in planktonic (SE-) samples. In contrast, clusters 1 and 2 predominates in planktonic (SE-) samples, comprising the majority of data points (Fig. 6B and C).

Poly-N-acetylglucosamine (PNAGs) are major components comprising biofilms, which are especially found in our biofilm strain (RP62A), ica operon, responsible for production of PNAG is completely absent in our planktonic strain (SE-) [45,49,50]. Furthermore, there are characteristic peaks in the absorption spectrum of PNAG at the wavelengths used in the measuring system [51]. From this it can be concluded that PNAG has a direct influence on the measured values in this range. Considering this information, we presume that cluster 4, exclusively revealed in biofilm (SE+) samples represent some structural component or a chemical group, which is highly prevalent in PNAGs. Looking in more detail at the spectral characteristics of this cluster, we can recognize a slight shift in the intensities of this cluster characterized by higher intensity at 2928 cm^−1^ (***ṽ***_***2∗***_) in comparison to 3352 cm^−1^ (***ṽ***_***3∗***_). Therefore, we can conclude that detection using FTIR yields a stronger signal at ***ṽ***_***2∗***_, corresponding to the CH_2_ group, compared to ***ṽ***_***3∗***_, which represents the OH and NH groups (Fig. 6E and F).

**Fig. 7:**
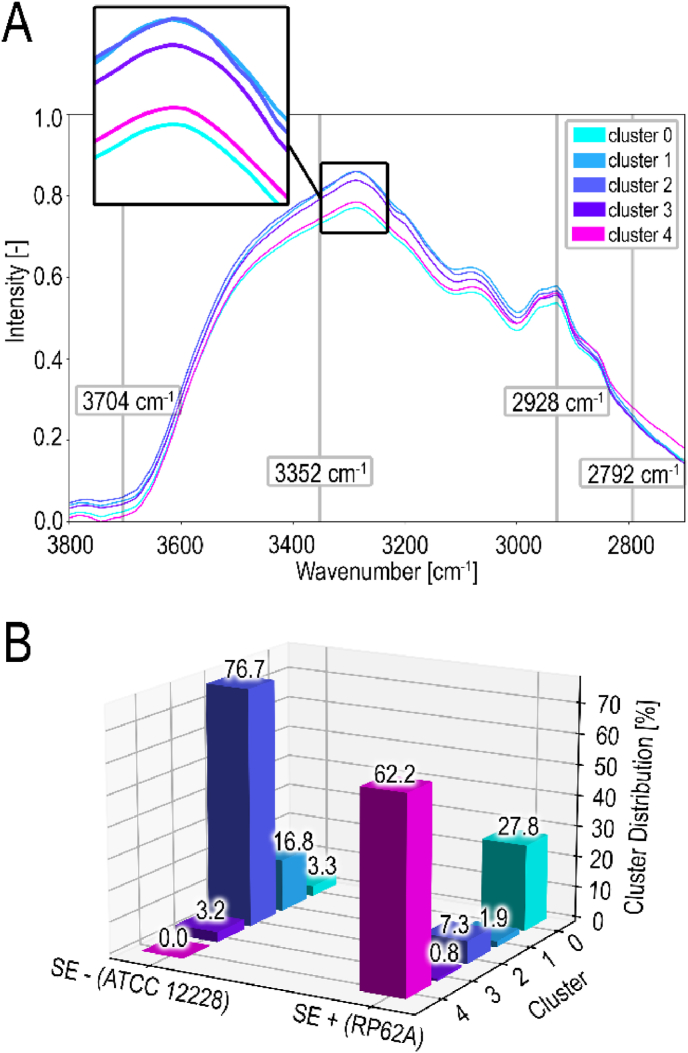
**Spectral correlation and quantification of all clusters revealed by FTIR.** All the different cluster sections for segmenting the biofilm, for all acquired FTIR scans, visualized in a mean spectrum for each cluster group (**A**). Percentage distribution across five clusters calculated for each strain (SE- and SE+; **B**).

The spectral correlation is constructed from average values of each cluster, which shows how the scatter plots and the clustering analysis relate to the FTIR spectrum of *S.epidermidis* (Fig. 7), representing the whole FTIR dataset. The overall shape and the peaks in FTIR spectrum of SE+ and SE-shown by Fig. 7A is consistent with the findings revealed by previous studies in microbial FTIR-spectroscopy [28,52].

It is visible that the cluster 4 shows low absorbance in the region of the NH-band (3350cm-1), which is consistent with location of cluster 4 coordinates, represented by magenta color (Fig. 6F). Cluster 0, represented by the cyan color, also exhibits lower absorbance, similar to cluster 4. However, based on the scatter plot data (Fig. 6E and F), cluster 0 does not stand out as a distinctive marker for biofilm (SE+) samples.

The FTIR data of all biofilm and planktonic samples were quantified in bar graphs as percentage distributions of each of the five clusters across SE+ and SE-strains separately (Fig. 7B). Thus, the cluster analysis of FTIR data indicated that predominantly cluster 4 serves as a valid marker for SE+ and can be used to differentiate biofilm by detecting the presence of *S. epidermidis* (RP62A)-biofilm.

### 3.4 Rapid detection of biofilm and planktonic strains using an innovative MIR system

**Fig. 8:**
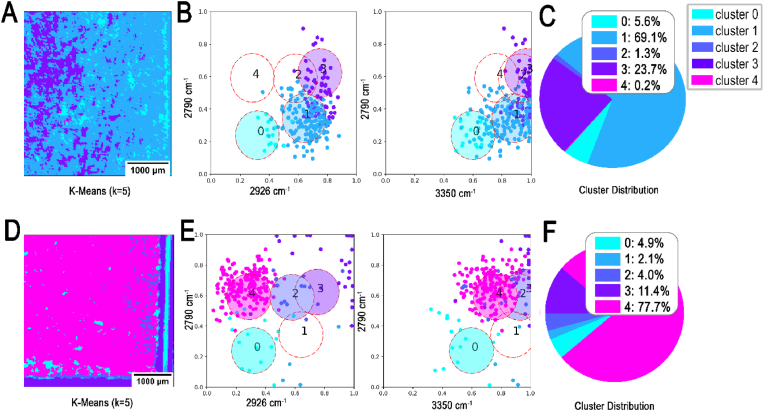
**K-means cluster analysis of biofilm and non-biofilm *S.epidermidis* samples processed by MIR**. K-means algorithm (k = 5) applied on SE- (**A, B, C**) and SE+ (**D, E, F**). Four-dimensional (2792 cm^−1^, 2928 cm^−1^, 3352 cm^−1^ and 3704 cm^−1^) input data as 2D arrays acquired with FTIR. Presenting the final clustering image obtained by K-means algorithm at convergence (**A, D**), two corresponding scatterplots for clustering visualization showing 10 % of the data (**B, E**) and a percentage distribution of the data within the cluster analysis (**C, F**).

Finally, the planktonic and biofilm samples were examined by the rapid enhanced MIR prototype recording at ***ṽ***_***1***_, ***ṽ***_***2***_, ***ṽ***_***3***_ and ***ṽ***_***4***_. The lasers installed in the MIR system provide more power on the specific wavenumber, than the broadband light source installed in the FTIR spectrometer. High power lasers increase the sensitivity and depth of detection. In Fig. 8, a similar color scheme can be seen in the analyzed MIR compared to FTIR data (Fig. 6). In addition, cluster 1 showed a significant shift in intensity with distribution of 69.1 % (Fig. 8A–C, SE-) between ***ṽ***_***2***_ and ***ṽ***_***3***_. This shift is characterized by increased intensity at ***ṽ***_***3***_ by about 0.3 AU (Fig. 8C) compared to 2.1 % in SE+ **(Fig. D-F**).

Presence of cluster 4, magenta color, was revealed in biofilm samples (SE+) processed by MIR scanner, with distribution of 78 % (Fig. 8 E and F), Contrary to cluster 1, the intensity at ***ṽ***_***3***_ was significantly higher with a shift of about 0.5 AU to the right. (Fig. 8F). It has been already mentioned that SE + biofilms predominantly consist of PNAGs, which is absent in planktonic (SE-) phenotypes [49,50]. However, despite lack of ECM production by planktonic strain (SE-), the adhesive characteristics of bacterial cells to the surfaces are still retained and mediated by substances such as polysaccharide adhesin (PS/A), which are present in SE-strain (ATCC 12228) [45,49,50]. Most likely cluster 1, resembles proteins of the planktonic cells which are attached to the slide surface through adhesins.

**Fig. 9:**
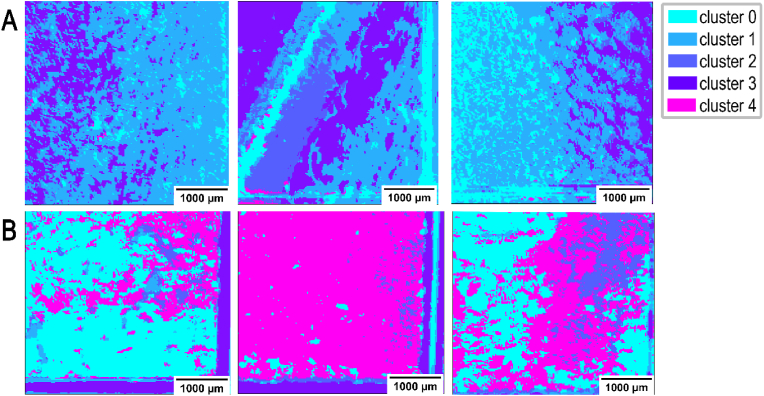
**Visualization of the comparison between biofilm and planktonic states acquired with MIR scanning.** Three representative images for illustration of K-means algorithm (k = 5) used in analysis of planktonic (SE-) (**A**) and biofilm SE+ (**B**) samples. Four-dimensional (2790 cm^−1^, 2926 cm^−1^, 3350 cm^−1^ and 3700 cm^−1^) input data as 2D arrays acquired with MIR.

The MIR data shows a similar color scheme compared to FTIR measurement but with visual higher contrast (Fig. 9), attributed to the greater power of MIR lasers at specific wavenumbers compared to the broadband light source in the FTIR system. Additionally, the optimized detection system for the narrower wavenumber range enhances sensitivity, yielding data with a depth of 0–64,000 counts in novel MIR, compared to 0 to 20,000 counts in FTIR.

### 3.5 Resolution comparison of the clustering data for biofilms

**Fig. 10:**
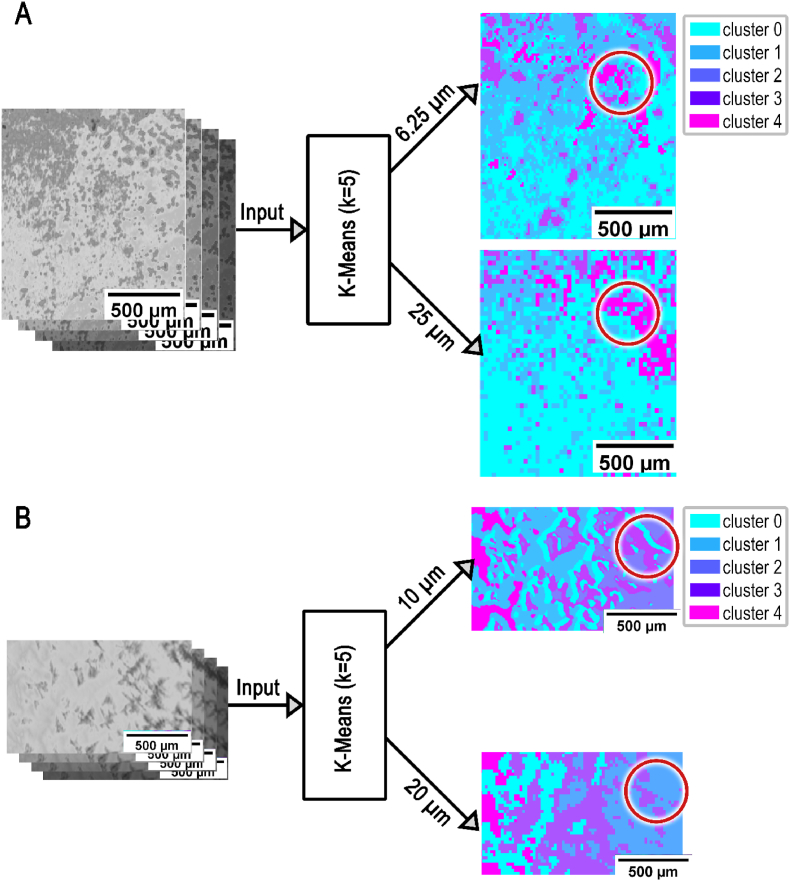
**Schematic of resolution improvements for FTIR and MIR scanning systems.** SE+ (RP62A) recorded and then clustered with FTIR (**A**) at a resolution of 25 μm compared to a resolution of 6.25 μm. Further MIR (**B**) at a resolution of 20 μm compared to a resolution of 10 μm. The K-means clustering (k = 5) was done using the wavenumbers 2790 cm^−1^, 2926 cm^−1^, 3350 cm^−1^, 3700 cm^−1^ as input according to chapter 2.7.

We used most optimal resolution in the assessment of the planktonic and biofilm samples. Measurements carried out by FTIR were performed with 6.25 μm resolution and by MIR with 10 μm resolution (Fig. 10). The sharpness of the image improves with resolution and the data can be better separated allowing to differentiate between small strictures. This affects distribution of clusters and increases number of data points.

Since the higher resolution can be used as standard in the commercially available FTIR (Fig. 10A), but not in the self-built MIR prototype, a possibility had to be created to generate higher resolution MIR scans (Fig. 10B). For this purpose, the methodology described in chapter 2.6 was used with the finer rasterization of spatial rastering up to 5 μm. This made it possible to improve the resolution of the scanning system by 100 %. The differences in resolution when examining biofilm are shown in Fig. 10.

Fig. 10A and B depict different sub-areas of a RP62A sample.

It has been demonstrated that higher resolution not only improves the clustering of details but also reassigns sub-areas to different segmentation classes (Fig. 10). The enhancement of resolution in MIR set-up was valuable in detecting and segmenting the clusters.

At lower resolution, the visualization of smaller sub-regions within the samples was inconsistent, precluding their inclusion in the clustering analysis. Moreover, the higher-resolution raw data allowed for significantly improved differentiation between larger morphological structures, particularly sharpening the edges of these structures. As the images in this study represent small sub-regions of the sample substrate, it should also be emphasized that the boundaries of chemical differences were not clearly resolved at lower resolution, further underscoring the importance of higher resolution for accurate morphological and chemical characterization.

### 3.6 Visualization of fixed biofilm after FTIR and MIR scanned slides

**Fig. 11:**
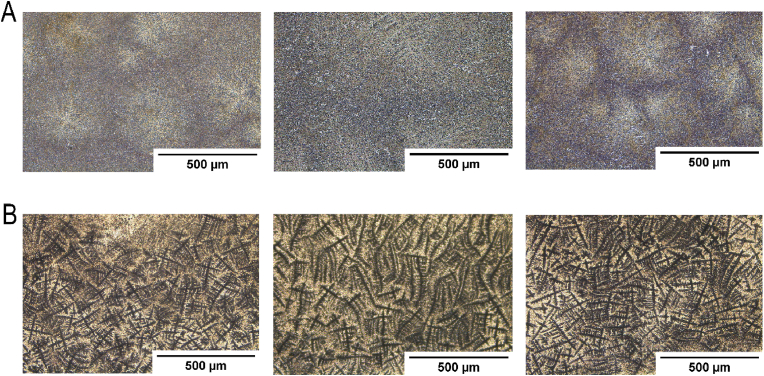
**Microscopic Comparison of Planktonic (ATCC 1228) and Biofilm (RP62A) Forms.** SE- (**A**) and SE+ (**B**) cultured for 72 h on silver coated glass slides were examined by bright field microscopy. About 10 images (n = 10) per slide were taken across different regions of the slides. Three slides per bacterial strain were analyzed. Images were taken using a LEICA microscope.

Next, we conducted a brief microscopic examination (Fig. 11) of biofilm-producing (SE+) and planktonic (SE-) cultured on silver-coated glass slides. To maintain identical conditions for bacterial culture, biofilm production, and fixation, we used the same samples that had been previously used for FTIR and MIR recordings.

Significant differences in the structural patterns of planktonic (SE-) and biofilm (SE+) were observed in various regions of the silver-coated glass slides under light microscopy (Fig. 11). The planktonic (SE-) strain does not produce biofilm [45]. Therefore, we did not expect to observe any visual patterns that can resample biofilm, and this expectation was confirmed after assessing multiple regions across each silver-coated glass slide, repeated for three slides. The planktonic samples exhibited a cold color tint, characterized by a pale light purple-blue hue. Although some round-shaped spheres were present, they did not display any prominent structural patterns (Fig. 11A). While some color variations were noted in the centers of these forms, the change was gradual, lacking in contrast. Additionally, the centers of the round forms featured a cross-like figure that appeared very blurry, without clear outlines, and was the lightest element in the image (Fig. 11A). The biofilm (SE+) samples (Fig. 11B), displayed a completely different appearance compared to the planktonic form. The biofilm samples exhibited a much warmer color tint, characterized by a beige-white-yellow background with a network of brown-black lines that crossed each other perpendicularly. These lines resembled bridge-like structures with branches, which were absent in the SE-samples. The bridges varied in length from approximately 50 μm–400 μm, with the branches being shorter than 50 μm (Fig. 11B). It is possible that the formation of these bridge-like structures corresponds to biofilm morphology.

## 4 Discussion

Periprosthetic Joint Infections (PJIs) are a major complication in joint replacement surgeries, often associated with biofilm formation on the surfaces of implants. Due to the high volume of patients, fast detection is essential in orthopedic surgery rooms. Rapid diagnosis with quick detection during surgery is essential for timely decision-making, as slower methods may fall short for immediate intraoperative management. There is definitely room for technological improvement. For example, even the fastest microbiology panels, such as BCID-GP and Verigene, take over an hour to identify pathogens and their drug resistance mechanisms [53]. In hospital settings, exploring even faster diagnostic approaches than BCID-GP would be more advantageous. Bacterial biofilms protect pathogenic microorganisms from both the host immune response and antibiotic treatments [9,10], making it critical for diagnostic approaches to address not only microbial detection but also the identification of biofilms.

Spectroscopy methods show considerable potential in clinical diagnostics, offering faster and more accurate alternatives for disease monitoring, including the detection of bacterial biofilms, which could be explored for optimizing treatment strategies. Substantial research has been conducted using Raman spectroscopy to characterize microbial signatures from various organisms [31,[54], [55], [56]]. Raman spectroscopy offers weaker but water-resistant signals, providing an advantage for studying biofilms, however IR spectroscopy uses less energetic excitation radiation [56]. While IR and Raman spectra are complementary, micro-Raman enables detailed analysis at the single-cell level, often enhanced by surface-enhanced Raman scattering (SERS) [54,57,58]. SERS requires careful sample preparation, as the signal depends on factors like nanoparticle size and environment [[58], [59], [60]]. Combined with optical microscopy, micro-Raman provides non-destructive, 3D analysis of biofilm components [56,61]. Nevertheless, IR spectroscopy still has the potential advantage over Raman methods, as it causes less disruption to the sample's native state compared to the aforementioned Raman spectroscopy methods. Although IR spectroscopy holds significant promise for biofilm research [[26], [27], [28]], its application in clinical diagnostics, particularly for periprosthetic joint infections, necessitates further optimization. To be clinically effective, the method must deliver rapid results from sample acquisition to analysis, while ensuring high sensitivity and accuracy. New detection techniques are always appreciated in clinical settings, provided they are cost-effective and save physicians' time.

In this study, we introduced a novel approach for the rapid spectral detection of bacterial biofilm using a novel mid-infrared (MIR) scanning system. This MIR approach eliminates the need for a 35-min pre-cooling period and manual liquid nitrogen filling, which are required for FTIR systems and can lead to suboptimal images due to slight deviations. In contrast, the prototype's detection mechanism is directly and electrically cooled, ensuring instant, reliable and consistent performance, which has already been tested in visualization of brain structures and of latent fingerprints [38,39]. When comparing our MIR approach to conventional Raman spectroscopy, several key differences emerge. MIR detects dipole moment changes (e.g., CH_2_, NH) and is fluorescence-free, whereas Raman spectroscopy is sensitive to non-polar bonds (e.g., C=C, C–H) but can be affected by fluorescence [35,62]. The MIR system, using targeted spectral bands, enables significantly faster measurements, while Raman provides deeper penetration but requires long acquisition times due to weak scattering signals [35]. Additionally, MIR benefits from strong absorption features, allowing efficient chemical clustering with fewer spectral points, whereas Raman requires high-power lasers and sensitive detectors [35,62,63]. These advantages of MIR primarily enhance its time and cost efficiency, enabling faster operation with minimal compromise to the quality of the analysis.

To demonstrate the comprehensive benefits and advantages of our MIR system, we conducted a comparative analysis against measurements obtained using a commercially available FTIR scanner. For the biofilm investigation, we utilized the clinically relevant methicillin-resistant *Staphylococcus epidermidis* strain RP62A (SE+) [11]. As a negative control, we used *S. epidermidis* ATCC 12228 (SE-), a non-infectious strain that does not form biofilms [45]. Although *E.coli* is often also used as a negative control in biofilm formation studies, SE-is more appropriate because of its retention of adhesion genes despite its lack of biofilm production [45]. This we also confirmed with crystal violet (CV) assay upon the comparison of *E.coli* and SE-read-outs after measuring absorbances. As expected, RP62A produced significantly stronger biofilms compared to the SE-control and *E.coli* as further control. It was found that *S. epidermidis* fully transitions to mature biofilm cell clusters after 72 h [46,47]. We have also confirmed with CV assay that after 72 h, biofilm production by RP62A was two times higher than after 24 h, therefore, to characterize biofilm-specific markers, we conducted FTIR and MIR recordings on biofilms cultivated for 72 h, comparing the phenotypes of RP62A (SE+) and ATCC 12228 (SE-). The chemical markers of RP62A and ATCC 12228 biofilms have been recently addressed in studies utilizing broad FTIR scanning, however the research question focused on the changes of biofilm over time using conventional markers [26].

For the comparison of the FTIR and MIR systems, measurements were taken at four specific wavenumbers: 2790 cm^−1^ (***ṽ***_***1***_), 2926 cm^−1^ (***ṽ***_***2***_), 3350 cm^−1^ (***ṽ***_***3***_), and 3700 cm^−1^ (***ṽ***_***4***_), focusing on a relatively narrow spectral range (∼3800–2800 cm^−1^). While this limited range excludes the detection of key biofilm components, such as extracellular DNA and certain proteins and polysaccharides that exhibit signals below 2800 cm^−1^, it allows for a focused analysis of the lipid region (CH_2_) typically detected at ***ṽ***_***2***_ and protein region (NH) detected at ***ṽ***_***3***_ [39,55].

The comparison demonstrates that using fewer wavenumbers yields representative results in both the commercial FTIR and self-built MIR systems for detecting biofilm markers, characterized by a presence of cluster 4. The results aligned with the microscopic analysis, revealing significant visual differences between the biofilm (SE+) and planktonic (SE-) samples. The biofilm (SE+) samples exhibited a unique morphology, characterized by bridge-like structures with branching patterns. This distinct morphology likely corresponds to cluster 4, as identified by FTIR and MIR detection. However, it holds potential for enhancing the differentiation between bacterial strains, as well as quantifying biofilm and bacterial load in a rapid manner suitable for clinical diagnostics. Further research is necessary to identify distinct clusters corresponding to specific bacterial strains.

Notably, the MIR system not only detected a biofilm marker but also identified a marker of the planktonic strain, represented by cluster 1. Additionally, given the faster acquisition time of the self-developed MIR system, this validates its potential for application in clinical diagnostics.

The EPS of SE+, which constitutes up to 90 % of the biofilm mass [1], is predominantly composed of poly-N-acetylglucosamine (PNAG) [15,49,50]. A key structural feature of PNAG is the glucosamine ring, characterized by strong –OH and N–H stretching vibrations. These N–H vibrations can overlap with the O–H stretch, given the 1:3 ratio of –NH to –OH groups in each N-acetylglucosamine monomer. The ***ṽ***_***3***_ region of the spectrum corresponds to areas where O–H and N–H groups exhibit broad, strong absorption bands, while ***ṽ***_***2***_ is typically associated with CH_2_ groups, which are characteristic of the lipid region [26,39,55]. Given the low lipid content in biofilms, it is unlikely that the shift of cluster 4 to higher intensity at ***ṽ***_***2***_, in biofilm (SE+) samples processed with FTIR, is due to lipids. The signal detected at ***ṽ***_***2***_ is more likely attributed to high presence of methylene (–CH_2_–) bridges formed during cross-linking reactions as a result of formaldehyde treatment for fixation.

Planktonic (SE-) bacteria are unable to produce biofilms but can still adhere to surfaces due to the retention of adhesive substances [45]. While SE + biofilms predominantly comprised of PNAGs, PNAGS are not exhibited by planktonic (SE-) phenotypes [49,50]. Nevertheless, planktonic (SE-) can still attach either individually or in small clusters on the slide surface. The CV assay shows that the adhered mass of SE-cells is 2.1 times lower than that of SE + cells, highlighting the reduced adhesion. Chemical signature in planktonic (SE-) samples were detected more effectively with MIR than with FTIR, due to its increased sensitivity. The high power of MIR lasers, however, enabled successful detection. The adhered structures, most likely corresponds to planktonic cells adhered to the slides, are represented by cluster 1. Results of the rapid MIR system revealed distinct intensity differences between clusters 1 and 4 at ***ṽ***_***2***_ and ***ṽ***_***3***_. Both clusters exhibited higher intensity at ***ṽ***_***3***_ than ***ṽ***_***2***_, indicating a greater presence of NH groups (amide A) compared to lipids. In planktonic samples, the CH_2_ groups at ***ṽ***_***2***_ likely originate from lipids in the bacterial cell wall, while in biofilm samples, the CH_2_ signal at ***ṽ***_***2***_ is attributed to methylene bridges formed during formaldehyde (FA) fixation. The stronger signal at ***ṽ***_***3***_ is consistently associated with NH groups in both planktonic and biofilm samples—likely from PNAGs in biofilms and proteins in planktonic cells. However, the precise chemical interpretation of these signatures is less critical due to some ambiguity. The most important aspect of rapid MIR detection is the clear distinction between the clusters, which serves as the key identifying feature.

**Fig. 12:**
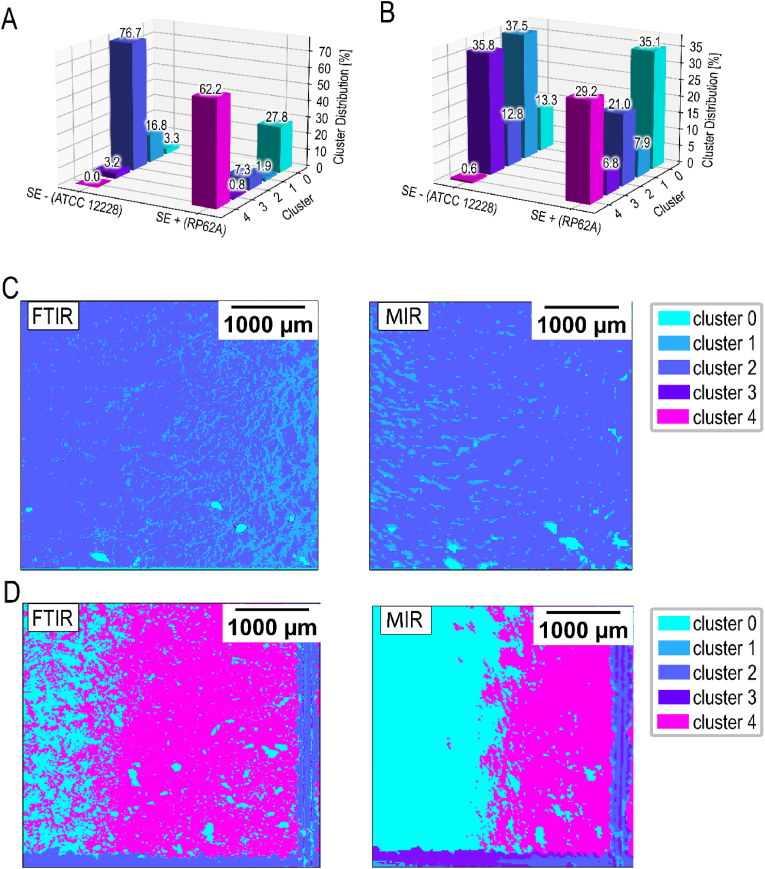
**Mean cluster distribution and summary of all findings comparing FTIR and MIR.** Mean cluster distribution for SE- (ATCC 12228) and SE+ (RP62A) in comparison for the acquired FTIR (**A**) and novel MIR (**B**) data. Panel (**C and D**) show a representative side-by-side comparison of FTIR and MIR data for the same measurement field in SE- (**C**) and SE+ (**D**).

In total, The MIR data exhibits a comparable color scheme to FTIR, but with significantly higher contrast. This increased contrast is attributed to the fact that the lasers in the MIR system can deliver more power at specific wavenumbers compared to the broadband light source used in the spectrometer. Additionally, the detection system is optimized for the narrower wavenumber range utilized, enhancing sensitivity within this range and providing a greater depth of data, with values from 0 to 64000, compared to the FTIR values ranging from 0 to 20000. An examination of the data allocation from the MIR and FTIR images reveals that the cluster groupings have similar distributions. In particular, the high percentage classification of samples originating from SE+ (RP62A) in cluster 4 is comparable with both devices (Fig. 12).

Enhancing the resolution in the MIR setup led to increased sensitivity, which favored detection of planktonic SE-. Higher resolution not only improves the clustering of detailed features but also reassigns sub-areas to different segmentation classes, enhancing overall accuracy. Additionally, finer resolution allows for more precise delineation of edge areas within clusters. Higher resolution also increases the number of data points per area, enhancing the performance of evaluation algorithms. Despite the higher resolution of FTIR increasing measurement time by a factor of 16, the MIR prototype only experiences a fourfold increase. This is due to differences in technical features/properties of the two used measuring techniques/devices [39]. Higher resolutions of possible medical devices lead to more accurate diagnostics, particularly for heterogeneous samples such as biofilms in periprosthetic joint infections. Additionally, higher resolution is crucial for accurately delineating the boundaries of abnormal or infected tissue regions, giving the MIR system an advantage over biosensors based on microbial electrochemical technologies (METs). While METs enable rapid online detection of biological or chemical oxygen demand (BOD) and (COD) sensors, reducing response times from days to hours compared to traditional kits, calibration and sensitivity remain significant challenges [64].

This study has several limitations that merit consideration. Specifically, an ideal comparison between surface biofilm and planktonic cells would involve using the same strain (RP62A) for both experimental conditions. However, due to the 72-h cultivation period selected for the biofilm formation, replicating the same conditions for planktonic cells was not feasible, as they would enter their death phase before reaching the desired experimental timeframe. Furthermore, despite the overall suitability of the MIR system for this measurement scenario biofilm detection, it should be noted that the resulting images differ from those produced by FTIR. Due to the variations in resolution, it is not feasible to employ calculation methods such as the Adjusted Rand Index (ARI) or Normalized Mutual Information (NMI) to obtain an assessment factor. Given the known effects of Staphylococcus-induced metal corrosion and the use of silver-coated glass slides for biofilm growth, it is possible that some of the bacteria generated patterns on the slides. Although a consistent layer of bacteria adhered to the slides was observed, it is possible that the cluster analysis and resulting data may not exclusively reflect biofilm structures. Furthermore, variations in clustering within individual scratches indicate potential inconsistencies in the ablation process. Outlier values are also assigned to clusters, suggesting the presence of artifacts in the data. The scratches were manually created using a scalpel. The bacterial or cell medium layer was applied as an ultrathin coating onto a slide covered with a few nanometer of silver. Manual ablation can significantly impact detection, as it remains uncertain which of the described layers were removed and which remained intact. Therefore, the scratches can just be used as spatial orientation, while the data points are to be seen as outlier values that have been assigned to a cluster group that is closest to these. Due to the chemical variability among bacterial strains, it remains uncertain whether the classification approach used in this study is universally applicable for distinguishing biofilm-forming from non-biofilm-forming bacteria. Further measurements with additional strains are necessary to confirm its generalizability.

## 5 Conclusion

In this study, we applied a custom-built MIR prototype for biofilm detection, with scanning features that target only four wavenumbers: 2790 cm^−1^ (***ṽ***_***1***_), 2926 cm^−1^ (***ṽ***_***2***_), 3350 cm^−1^ (***ṽ***_***3***_), and 3700 cm^−1^ (***ṽ***_***4***_). This narrower spectral range (∼3800–2800 cm^−1^), which covers less than one-third of the broad FTIR region (∼750–4000 cm^−1^), was sufficient to differentiate between biofilm and non-biofilm phenotypes. While scanning of the broader spectral range typically provides more detailed information, our reduced range still effectively captured the necessary distinctions. This was possible to achieve via a self-built MIR by concentrating laser power on these targeted wavenumbers (***ṽ***_***1***_*,* ***ṽ***_***2***_*,* ***ṽ***_***3***_*,* ***ṽ***_***4***_), as opposed to the even distribution of energy required for broadband scanning in commercial FTIR devices. The data obtained with the self-built rapid MIR scanner was validated by a commercially available FTIR scanner, demonstrating the effectiveness of innovative MIR technology.

We conclude that with the rapid MIR device, we can achieve better and faster biofilm differentiation, notably in seconds, but also within the distribution of the clusters compared between biofilm and non-biofilm producing *S.epidermidis* strains. It has been shown that interdisciplinary collaborations, which include biological and physical sciences, engineering and medicine can facilitate innovative solutions and transfer effective translation of research funding into practical clinical applications. The secondary achievement is integration of multidisciplinary knowledge for contribution in technological optimization. Continuous technological optimization in both processing speed and cost is absolutely essential for managing healthcare procedures effectively and addressing the numerous challenges faced by modern healthcare systems. By improving the efficiency and affordability of diagnostic tools, treatment methods, and patient management systems, medical care can better cope with increased patient capacity, ensure timely diagnoses and treatments, and ultimately enhance patient outcomes. This ongoing optimization is critical to keeping pace with the growing demands on healthcare resources and ensuring that high-quality care is accessible to all patients in the effective times.

## CRediT authorship contribution statement

**Björn van Marwick:** Writing – original draft, Validation, Methodology, Investigation, Conceptualization. **Tatyana N. Sevastyanova:** Writing – original draft, Visualization, Methodology, Investigation, Conceptualization. **Felix Wühler:** Visualization, Software, Formal analysis, Data curation. **Barbara Schneider-Wald:** Writing – review & editing, Methodology. **Cornelia Loy:** Visualization, Investigation. **Sascha Gravius:** Resources, Investigation, Funding acquisition, Conceptualization. **Matthias Rädle:** Resources, Investigation, Funding acquisition, Conceptualization. **Andreas Schilder:** Writing – review & editing, Resources, Investigation.

## Declaration of competing interest

The authors declare the following financial interests/personal relationships which may be considered as potential competing interests: Bjoern van Marwick reports financial support was provided by 10.13039/100021130Federal Ministry for Economic Affairs and Climate Action. Bjoern van Marwick reports financial support was provided by Baden-Württemberg Ministry of Science, Research and Culture. Bjoern van Marwick reports financial support was provided by Center for Mass Spectrometry and Optical Spectroscopy (CeMOS). Bjoern van Marwick reports a relationship with Center for Mass Spectrometry and Optical Spectroscopy (CeMOS) that includes: employment. If there are other authors, they declare that they have no known competing financial interests or personal relationships that could have appeared to influence the work reported in this paper.

This Project is supported by the 10.13039/100021130Federal Ministry for Economic Affairs and Climate Action (BMWK) on the basis of a decision by the German Bundestag.

The article processing charge was funded by the Baden-Württemberg Ministry of Science, Research and Culture and the Center for Mass Spectrometry and Optical Spectroscopy (CeMOS) in the funding program Open Access Publishing.

We thank the graduate program Perpharmance BW6_07, Ministry of Science, Research and Culture (MWK), Baden-Württemberg for financial support.

## Data Availability

Data will be made available on request.
